# COVID-19 Impact on Cerebrospinal Fluid Diversion: A Single Institution Experience

**DOI:** 10.7759/cureus.26578

**Published:** 2022-07-05

**Authors:** Shelly Sharma, Klaudia Dziugan, Ava Kucera, Sandi Lam, Michael DeCuypere

**Affiliations:** 1 Division of Pediatric Neurosurgery, Ann and Robert H. Lurie Children's Hospital of Chicago, Chicago, USA; 2 Department of Neurological Surgery, Northwestern University Feinberg School of Medicine, Chicago, USA

**Keywords:** neurological surgery, pediatric, hydrocephalus, csf diversion, covid-19

## Abstract

Introduction: Several studies have demonstrated that the absolute numbers of select surgical interventions for myocardial infarction, stroke, and appendicitis decreased during the COVID-19 pandemic, possibly due to overall decreased hospital presentation. We sought to identify if this pattern was also true for children with hydrocephalus and cerebrospinal fluid (CSF) diversion procedures. We hypothesized that there would be a detectable decrease in CSF diversion procedures performed during the COVID-19 pandemic, as compared to a pre-COVID-19 baseline.

Methods: A chart review of all patients that underwent a CSF diversion procedure from March 2019 to February 2021 was performed at Ann and Robert H. Lurie Children’s Hospital of Chicago. The pre-COVID-19 period was defined as March 2019 to February 2020 and the COVID-19 pandemic period was defined as March 2020 to February 2021. CSF diversion procedures included endoscopic third ventriculostomy (ETV), ETV/choroid plexus cauterization (CPC), initial shunt placement, shunt removal/replacement, shunt revision, and temporization procedures. Data included gender, race, ethnicity, insurance type, etiology of hydrocephalus, type of procedure, and whether the procedure was performed due to infection.

Results: Overall, there was no significant difference in the absolute number of CSF diversion procedures performed when comparing the pre-COVID-19 and COVID-19 periods (244 and 238, respectively). Furthermore, there was no observed difference in the gender, ethnicity, or insurance status of children undergoing a CSF diversion procedure. There was, however, a significant increase in the number of procedures performed due to infection during the COVID-19 pandemic at our institution (p = 0.04).

Conclusion: Unlike several other surgical conditions during the COVID-19 pandemic, a statistically significant change in CSF diversion procedures was not observed at our institution. The increased number of procedures for infection at our institution is likely multifactorial and will be investigated further.

## Introduction

As the United States entered the COVID-19 pandemic at the beginning of March 2020, a sudden decline in surgical cases, such as orthopedic and breast surgery cases [1], was seen not only in the United States, but in Great Britain [2], Jordan [3], and Italy [4]. Many studies released between late 2020 and early 2021 noted a decrease in outpatient procedures and inpatient admissions. Furthermore, when surgical case numbers were compared between early to mid-2020 to 2018 and 2019, a decrease in surgical procedures was observed. Duarte et al. reported a decrease of 17.8% in vascular surgeries performed between February and June 2020 when compared to 2018 and 2019 [5]. Ultimately, there was a demonstrated decrease in the absolute numbers of select surgical interventions for myocardial infarctions [6], stroke (with longer door-to-needle time) [7], and appendicitis [8] during the COVID-19 pandemic, possibly due to overall decreased hospital presentation. Alongside many surgical specialties, adult neurosurgical procedures also declined early during the pandemic [9,10].

Pediatric surgical volume had a noted reduction in the United States between 2019 and 2020 [11,12]. One study found a decrease in 55% of pediatric surgeries between March to May 2019 and 2020. However, the differences in a number of cases did vary among surgical departments with otolaryngology seeing a drop of 76% compared to a reduction of 14% in neurosurgery.

We sought to identify if this pattern was also true for children with hydrocephalus and cerebrospinal fluid (CSF) diversion procedures at Lurie Children’s Hospital of Chicago. We hypothesized that there would be a detectable decrease in CSF diversion procedures performed from March 2020 to February 2021 (the COVID-19 pandemic), as compared to a pre-COVID-19 baseline. This article was previously presented as an oral presentation at the 17th WFNS World Congress of Neurosurgery Meeting on March 14, 2022.

## Materials and methods

Following institutional review board (IRB) approval (IRB # 2021-4375), a retrospective chart review of all patients that underwent a CSF diversion procedure from March 2019 to February 2021 was performed at Ann and Robert H Lurie Children's Hospital of Chicago. The pre-COVID-19 period was defined as March 2019 to February 2020 and the COVID-19 pandemic period was defined as March 2020 to February 2021, from the start of the Illinois statewide stay at home mandate to the first year of the pandemic. CSF diversion procedures included included endoscopic third ventriculostomy (ETV), ETV/choroid plexus cauterization (CPC), initial shunt placement, shunt removal/replacement, shunt revision, and temporization procedures. Data included gender, race, ethnicity, insurance type, etiology of hydrocephalus, type of procedure, and whether the procedure was performed due to infection. All patients from the COVID-19 pandemic period had their COVID-19 status prior to surgery noted as well. The primary outcome measure was to compare the absolute number of CSF diversion procedures performed before and after the peak of COVID-19. Secondary outcomes focused on the differences in an absolute number of procedures due to infection and acuity of cases, as determined by the neurosurgeon and noted in the operative, whether urgent (within 24 hours) or elective (outside of 24 hours). Differences in categorical variables were analyzed using a chi-square test and significance was determined with a p-value <0.05.

## Results

**Figure 1:**
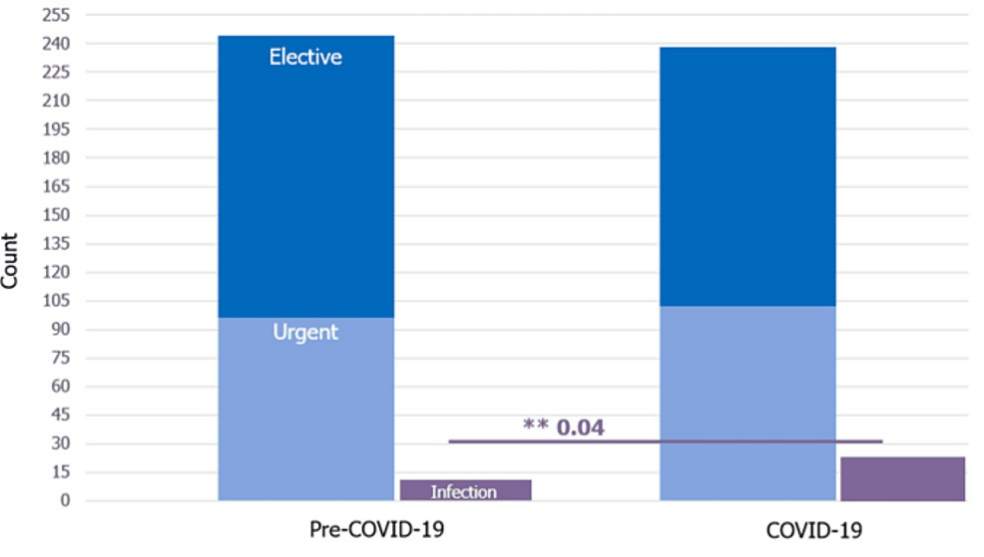
Surgical volume prior to and during COVID-19, urgent vs elective and infection ** significance at p-value <0.05

CSF diversion procedures represented 29% of 843 surgeries performed in the division of neurosurgery between March 2019 and February 2020 (pre-COVID-19). Following the COVID-19 pandemic, 31% of all 777 surgeries were CSF Diversion procedures (Figure 1). As shown in Table 1, there was no significant difference in the absolute number of CSF diversion procedures performed when comparing the pre-COVID-19 and COVID-19 periods (244 and 238, respectively) (p-value=0.38). Furthermore, there was no observed difference in the gender, race, ethnicity, or insurance status of children undergoing a CSF diversion procedure.

**Table 1:** CSF diversion procedures data PHHP: Post-hemorrhagic hydrocephalus of prematurity, ETV: Endoscopic third ventriculostomy, CPC: Choroid plexus cauterization

	Pre-COVID-19	COVID-19	P-value
All Procedures Done	843	777	
Total	244 (29%)	238 (31%)	0.38
Gender			0.41
Female	126 (52%)	114 (48%)	
Male	118 (48%)	124 (52%)	
Race			
Asian	5 (2%)	14 (6%)	
Black	49 (20%)	45 (19%)	
White	128 (52%)	140 (59%)	
Other	59 (24%)	36 (15%)	
Unknown	3 (1%)	3 (1%)	
Ethnicity			0.91
Hispanic	55 (23%)	52 (22%)	
Not Hispanic	190 (77%)	186 (78%)	
Insurance			0.71
Private	111 (45%)	104 (44%)	
Public	133 (55%)	134 (56%)	
Etiology			
Aqueductal Stenosis	8 (3%)	13 (5%)	
Benign Tumor	28 (11%)	18 (8%)	
Congenital	15 (6%)	36 (15%)	
Malignant Tumor	23 (9%)	28 (12%)	
Myelomeningocele	54 (22%)	39 (16%)	
Post-Infectious	16 (7%)	9 (4%)	
PHHP	70 (29%)	53 (22%)	
Trauma	1 (0.4%)	3 (1%)	
Other	29 (12%)	39 (16%)	
Procedure			
ETV, ETV/CPC	17 (7%)	34 (14%)	
Initial shunt placement	43 (18%)	34 (14%)	
Shunt removal/replacement	41 (17%)	65 (27%)	
Shunt revision	126 (52%)	94 (39%)	
Temporization	17 (7%)	11 (5%)	
Procedures for Infection	13 (5%)	25 (11%)	0.04
Elective vs Urgent			0.43
Elective Cases	148 (61%)	136 (57%)	
Urgent Cases	96 (39%)	102 (43%)	

There was, however, a significant increase in the number of procedures performed due to infection during the COVID-19 pandemic at our institution (p = 0.04). During the COVID-19 pandemic period, the most common infectious cause was Staphylococcus epidermidis (44%) followed by Propionibacterium acnes (12%) (Figure 1). Prior to the pandemic, the majority of CSF diversion procedures due to infection were associated with negative cultures (38%), with elevated cell counts demonstrating an underlying infection. Of the patients who did have a procedure performed due to infection during the pandemic, none were COVID-19 positive. Only four (1.6%) patients were COVID-19 positive at the time of their urgent procedures.

Although the result did not reach significance, there was a trend in more urgent procedures and a decrease in elective procedures performed between the pre-COVID-19 period and during COVID-19. Overall, 61% and 57% of CSF diversion procedures were elective prior to the pandemic and during the pandemic, respectively. The number of urgent procedures increased by 4% during the COVID-19 pandemic.

## Discussion

When the COVID-19 pandemic began in Illinois in March of 2020, patient and provider safety became a top priority at Lurie Children’s Hospital. All providers were given access to proper PPE, including fitted N95s, face shields, and surgical masks. Furthermore, from March 13, 2020, all patients were required to have a COVID-19 (SARS-CoV-2) test prior to their scheduled operation. For those who needed to undergo urgent surgeries with a positive COVID-19 test, special precautions were taken to maximize the safety of all staff involved in the operation, representing a commitment to patient care and the providers and hospital staff.

**Figure 2:**
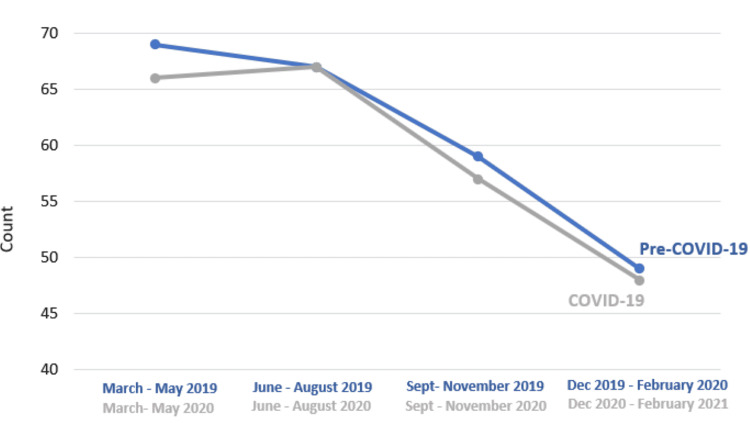
Surgical volume prior to and during COVID-19 pandemic by months

During the COVID-19 pandemic, there was a decrease in overall neurosurgical cases from 843 to 777 consistent with other pediatric surgical institutions [11,12]. Prior to the COVID-19 pandemic, the months of March to May had the highest surgical volume with a steady decrease into the winter months. While this trend for all surgical cases was lost during the pandemic, it was upheld for all CSF Diversion Procedures with the absolute numbers and percentages being comparable between the time periods (Figure 2). Unlike other institutions, our study did not find a statistically significant difference in the number of surgeries performed for CSF diversion during the COVID-19 pandemic, likely since CSF diversion for the treatment of hydrocephalus is typically not elective. Timely intervention is indicated in the event of hydrocephalus, increased intracranial pressure, and symptomatic shunt malfunction for shunted hydrocephalus. Our CSF diversion case data paralleled that noted by Wali et al. from UC San Diego Health, an institution that at the start of the COVID-19 pandemic put in place an OR triage plan where all procedures had to be approved by department chairs before being added to the OR schedule [13]. Gundi et al. noted that while there was a decrease in the frequency of elective surgeries during the pandemic for digestive, neonatal, urologic, and oncology cases, the frequency of emergency cases was similar [14]. While we observed a slight decrease in elective procedures and a small increase in procedures deemed urgent, these differences were not statistically significant (Figure 1). We show that the COVID-19 pandemic did not deter families from coming to the clinic, scheduling elective procedures, or visiting the emergency department to avoid further deterioration of their child’s condition in the setting of hydrocephalus. As other studies have emphasized, neurosurgical care and procedures are of essential manner: delay may lead to negative long-term outcomes [13,15].

A finding of interest is the observed increase in procedures performed due to bacterial infection during the COVID-19 period. The difference between pre-COVID-19 and COVID-19 rate of surgeries done for shunt infection is statistically significant (Figure 1, Table 1). This number is not reflective of shunt infections of our hospital: rather, it is the surgeries done for the indication of shunt infection. The reason for this observation may be multifactorial. Health system forces during the first wave of COVID-19 may have shaped patterns of care for pediatric patients in our area. For instance, there were increasing need for adult COVID-19 units. Conversion of hospital units to adult COVID-19 units correlated with closures or decreases in the number of pediatric hospital beds in our urban area. These market changes may result in the transfer of patients or the flow of patients seeking to come to our freestanding tertiary care center for children, which remained continuously open. The ramifications of such health system changes during this time are beyond the scope of this study and would warrant further exploration with another dataset. Staphylococcus epidermidis, a microorganism commonly found on human skin, has been previously identified in the literature as contributing widely to shunt infections due to its biofilm development [16], which was the most common cause of infection in our cohort during the COVID-19 pandemic. None of our patients who had a procedure done due to infection tested COVID-19 positive. Thus, this provides some evidence that the increase in infections may have been incidental.

This study has several limitations, including the inherent limited observational nature of retrospective chart review. The thought process of families in terms of decision-making of timing and need for seeking care is not elucidated well. The timing of onset of symptoms or concern to presentation and surgery is difficult to quantify. Relative delay in care, if present, is not known. Furthermore, we analyzed a subset of pediatric neurosurgical procedures, CSF diversion. We cannot generalize that other categories of pediatric neurosurgical procedure, such as brain tumor resections, would have similar results when comparing the number of procedures pre-COVID-19 and during COVID-19.

## Conclusions

The COVID-19 pandemic brought drastic changes to healthcare. Institutions saw a decrease in surgical volume as new protocols were established due to the pandemic. Unlike several other surgical conditions during the COVID-19 pandemic, a statistically significant change in CSF diversion procedures was not observed at our institution. The increased number of procedures for infection at our institution is likely multifactorial and will be investigated further.

## References

[1] Meredith JW, High KP, Freischlag JA (2020). Preserving elective surgeries in the COVID-19 pandemic and the future. JAMA.

[2] Sugand K, Park C, Morgan C (2020). Impact of the COVID-19 pandemic on paediatric orthopaedic trauma workload in central London: a multi-centre longitudinal observational study over the "golden weeks". Acta Orthop.

[3] Ennab RM, Ibdah RK (2021). The impact of COVID-19 on surgical practice in Jordan during the second outbreak: a survey. Ann Med Surg (Lond).

[4] Rocco B, Sighinolfi MC, Sandri M (2021). The dramatic COVID 19 outbreak in Italy is responsible of a huge drop of urological surgical activity: a multicenter observational study. BJU Int.

[5] Duarte A, Gouveia E Melo R, Lopes A, Rato JP, Valente J, Pedro LM (2021). Lessons learned from the impact of the COVID-19 pandemic in a vascular surgery department and preparation for future outbreaks. Ann Vasc Surg.

[6] Ruparelia N, Panoulas V (2020). The missing acute coronary syndromes in the COVID-19 era. Ther Adv Cardiovasc Dis.

[7] Xu X, Xiao Y, Li J (2022). Decrease in intravenous thrombolysis and poor short-term functional prognosis for acute ischemic stroke during the COVID-19 pandemic. J Neurol.

[8] Scheijmans JC, Borgstein AB, Puylaert CA (2021). Impact of the COVID-19 pandemic on incidence and severity of acute appendicitis: a comparison between 2019 and 2020. BMC Emerg Med.

[9] Patel PD, Kelly KA, Reynolds RA (2020). Tracking the volume of neurosurgical care during the Coronavirus Disease 2019 pandemic. World Neurosurg.

[10] Khalafallah AM, Jimenez AE, Lee RP (2020). Impact of COVID-19 on an academic neurosurgery department: the Johns Hopkins experience. World Neurosurg.

[11] Mattingly AS, Rose L, Eddington HS, Trickey AW, Cullen MR, Morris AM, Wren SM (2021). Trends in US surgical procedures and health care system response to policies curtailing elective surgical operations during the COVID-19 pandemic. JAMA Netw Open.

[12] Utria AF, Javid PJ, Chen J, Rice-Townsend SE (2021). Impact of COVID-19 on procedure volume at a tertiary pediatric hospital. Am J Surg.

[13] Wali AR, Ryba BE, Kang K (2021). Impact of COVID-19 on a neurosurgical service: Lessons from the University of California San Diego. World Neurosurg.

[14] Gunadi Gunadi, Idham Y, Paramita VM, Fauzi AR, Dwihantoro A, Makhmudi A (2020). The impact of COVID-19 pandemic on pediatric surgery practice: a cross-sectional study. Ann Med Surg (Lond).

[15] Bajunaid K, Alatar A, Alqurashi A (2020). The longitudinal impact of COVID-19 pandemic on neurosurgical practice. Clin Neurol Neurosurg.

[16] Gutierrez-Murgas Y, Snowden JN (2014). Ventricular shunt infections: immunopathogenesis and clinical management. J Neuroimmunol.

